# New Prognostic and Predictive Markers in Head and Neck Tumors

**DOI:** 10.1155/2016/2850502

**Published:** 2016-02-16

**Authors:** Monica Cantile, Francesco Longo, Gerardo Botti, Franco Fulciniti

**Affiliations:** ^1^Pathology Unit, Istituto Nazionale Tumori Fondazione “G. Pascale”, IRCCS, 80131 Naples, Italy; ^2^Head and Neck Surgical Oncology Unit, Istituto Nazionale Tumori Fondazione “G. Pascale”, 80131 Naples, Italy; ^3^Clinical Cytopathology Service, Institute of Pathology, Locarno, Switzerland

Head and neck cancers include a large and heterogeneous group of tumors with a very variable prognosis. These tumors arise from the squamous epithelial lining of the oral cavity, oropharynx, nasopharynx, hypopharynx, and larynx but include also lesions that arise from other anatomical sites, including the salivary glands, the mucosa of respiratory sinuses, thyroid, skin, and orbit. Sometimes, also cerebral tumors are included in this clinic-pathological chapter.

The molecular mechanisms associated with the pathogenesis and evolution of these diseases are poorly understood, although some recent indications, from gene expression profiling studies, suggested a series of well-characterized and new biomarkers able to diagnose and predict behavior and sensitivity to treatment of head and neck cancers.

The expression analysis of these biomarkers associated with histomorphological data could then provide the oncologist with the opportunity to create a proper stratification of patients for customized therapies.

In this special issue the authors contributed to highlighting the appearance and the relative importance of new prognostic markers and of innovative surgical approaches in the prognostic determinism of several head and neck tumors. In particular, T. Ius et al. analyzed factors influencing the tumor recurrence (TR) in a cohort of adult patients with an initial diagnosis of insular Low-Grade Gliomas that underwent a second surgery, without any adjuvant treatments between surgeries, showing that the extent of surgical resection (EOR) at first surgery represents the major predictive factor for TR. Another interesting study by V. Uloza et al. showed the first morphological and morphometric characterization of laryngeal squamous cell carcinoma on an animal model. Regarding the definition of new prognostic biomarkers, M. Tang et al. highlighted that macrophage inflammatory protein-3 alpha (MIP-3*α*) and cystatin A could represent valuable prognostic markers in nasopharyngeal carcinoma. Moreover, E. Lakiotaki et al. analyzed the clinical significance of cannabinoids CB1 and CB2 in thyroid lesions, underlining especially the usefulness and therapeutic potential of CB2. Finally, M. Hühns et al. showed that HHV-8 and EBV infection are not associated with salivary gland tumors, while HPV infection may play an important role in development and progression of these tumors.

We hope that the information included in this special issue can contribute to understanding of mechanisms associated with the pathogenesis and evolution of head and neck tumors.

*Monica Cantile* *Francesco Longo* *Gerardo Botti* *Franco Fulciniti*

